# 356. The Role of Procalcitonin in Antimicrobial Stewardship Among Cancer Patients Admitted with COVID-19

**DOI:** 10.1093/ofid/ofab466.557

**Published:** 2021-12-04

**Authors:** Hiba Dagher, Anne-Marie Chaftari, Ray Y Hachem, Ying Jiang, Alexandre Malek, Natalie J Dailey Garnes, Jovan Borjan, Victor Mulanovich, Issam I Raad

**Affiliations:** 1 UT MD Anderson Cancer Center, Houston, TX; 2 The University of Texas MD Anderson Cancer Center, Houston, TX; 3 MD Anderson Cancer Center, Houston, TX; 4 University of Texas MD Anderson Cancer Center, Houston, TX

## Abstract

**Background:**

Procalcitonin (PCT) has been used to guide antimicrobial therapy in bacterial infections. With the wide spread use of empiric use of antibiotics in cancer patients admitted with COVID-19 disease, we aimed to evaluate the role of PCT in decreasing the duration of empiric antimicrobial therapy among cancer patients admitted with COVID-19.

**Methods:**

We conducted a retrospective study of cancer patients admitted to MD Anderson Cancer Center who had a PCT test done within 72 hours of admission following their COVID-19 diagnosis between March 1, 2020 and June 6, 2021. Patients were divided into 2 groups of PCT < 0.25 ng/mL and PCT >=0.25 ng/mL. We assessed pertinent cultures including blood and respiratory, as well as antibacterial use and duration of empiric antibacterial therapy.

**Results:**

We identified 544 patients with a median age of 62 years (range, 14-93). There were 312 (57%) patients that had at least one culture obtained from a sterile or infected site within 7 days following admission. None of the patients who had PCT< 0.25 had a positive culture whereas 41/111 (37%) patients with PCT >= 0.25 had at least one positive culture [P< 0.0001]. Among the 373 patients who had a PCT < 0.25, 129 (35%) patients received more than 72 hours of IV antibiotics compared to 87/171 (51%) among patients with PCT >=0.25 [P= 0.0003].

**Conclusion:**

These results confirm the correlation between a PCT level greater than 0.25 and a documented bacterial infection. Furthermore, procalcitonin could be useful in enhancing antimicrobial stewardship in cancer patients with COVID-19 by reducing the duration of antimicrobial therapy beyond the initial empiric 72 hours until PCT results become available.

**Disclosures:**

**Natalie J Dailey Garnes, MD, MPH**, **AlloVir** (Other Financial or Material Support, collaborator on research protocol)

